# Product of mobility and lifetime of charge carriers in CdTe determined from low-frequency current fluctuations

**DOI:** 10.1038/s41598-024-51541-6

**Published:** 2024-01-09

**Authors:** J. Toušek, J. Toušková, I. Křivka

**Affiliations:** Faculty of Mathematics and Physics, Charles University, 182 00 Prague 8, Czech Republic

**Keywords:** Materials science, Physics

## Abstract

The model, which clarifies the low-frequency fluctuations of the current flowing in CdTe sample, makes it possible to determine the product of the mobility and lifetime of the charges in mentioned semiconductor. This model, with general validity for semiconductors, is based on the interaction of shallow traps with the valence or conduction band. As a result of the action of these centers, current fluctuations appear, the mean amplitude of which increases linearly with the inverse value of the frequency. It was found that the slope of this dependence is proportional to the product of mobility *µ* and a constant which is common to all shallow traps and which is denoted by the symbol *a.* The lifetime of charges located on shallow traps varies according to the relationship *τ* = *a*/*f* and for *f*_min_ it acquires a maximum value of *τ*_*max*_*,* which agrees with the stationary lifetime. For the p-CdTe crystalline semiconductor the mobility-lifetime product *µ*_*p*_*τ*_*p*_ = (6.6 ± 0.3) × 10^–7^ cm^2^V^–1^was obtained. Similar study of n-type CdTe showed *µ*_*n*_*τ*_*n*_ = (7.5 ± 0.3) × 10^–8^ cm^2^V^–1^.

## Introduction

Measured CdTe samples were found to generate fluctuations when DC current is passed through them. This effect can be compared to the sound produced when someone strikes the strings of a musical instrument. However, CdTe samples generate mostly very low frequencies outside the range of human audibility. Low-frequency electrical fluctuations whose power amplitude increases linearly with the inverse of the frequency are often referred to as "1/f noise" or flicker noise. This effect was first published by Johnson^1^ in 1925. After that, it became the subject of many papers and is also mentioned in book publications^2^. We have also previously shown that a direct current after passing through a polyaniline sample shows a well-reproducible low-frequency fluctuation of the current, the mean value of which has a linear course against the inverse of the frequency^3^. In this paper, we use the mean value of the current fluctuation and therefore do not use the term noise. We proposed a model based on the idea of the interaction of traps with the valence or conduction band. The interaction is characterized by the lifetime *τ*. The trapping of charges to the bandgap levels and release into the band causes the carrier concentration to fluctuate in the DC current, thereby modulating it. This leads to the linear dependence of the mean value of the current on 1/frequency. The slope of this dependence contains the *µτ* product.

## Theory and experiment

**Figure 1:**
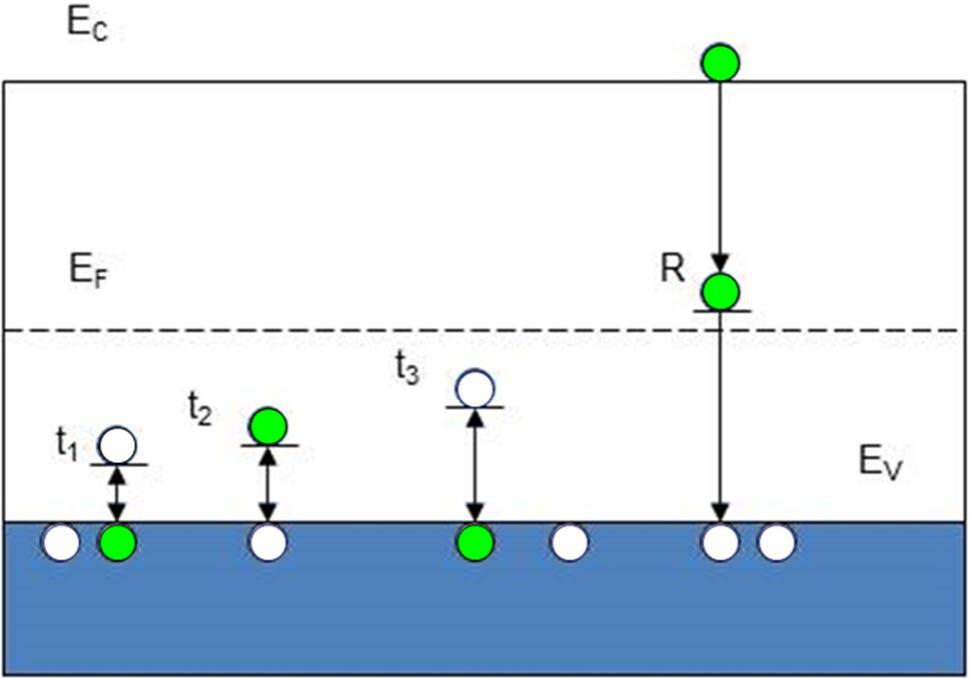
Band diagram of a p-type semiconductor with an example of processes leading to current fluctuations. Levels *t*_*1*_, *t*_*2*_*, t*_*3*_ represent the traps that are in equilibrium with the valence band, *R* is a deep center. Electrons (filled circles) can be thermally excited to trap *t*_1,_ and *t*_3_. This corresponds to the excitation of holes (open circles) from these traps into the valence band. In contrast, the transition of an electron from the *t*_2_ trap to the valence band can be considered as hole capturing at *t*_2_. The deep R level is filled by a different mechanism.

**Figure 2:**
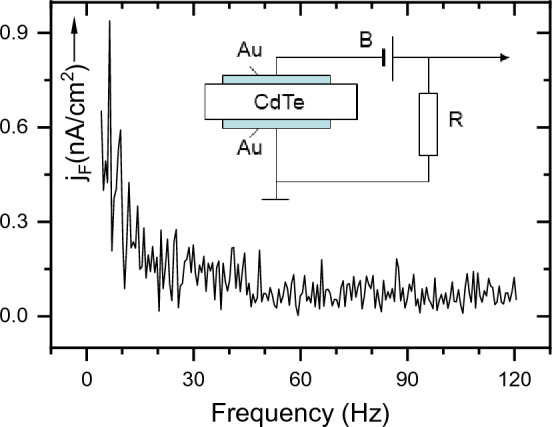
Fluctuation current density *j*_*F*_ in p-CdTe crystalline sample is plotted in the range of 4–112 Hz with the step 0.6 Hz. The inset picture shows the experimental arrangement: B is the battery; R is the load resistor, from which the alternating signal for the amplifier is taken.

An explanation of the formation of direct current fluctuation is shown in Fig. 1. We outline a model that attempts to show that the peaks of fluctuations (See Fig. 2) originate from the interaction between the band gap levels and the valence band. Traps *t*_1_, *t*_2_, *t*_3_…are levels in the band gap that are said to be in equilibrium with the valence band when the number of captured holes equals the number excited in the valence band. As deduced below, this interaction leads to the linearity of the mean value of the fluctuation current against 1/*f*. In contrast, the hole trapped at the deep *R* level is not excited back and recombines with an electron from the conduction band.

In this work, the low-frequency current fluctuations were studied on non-doped crystalline p-type cadmium telluride (CdTe) material of conductivity 1.4 × 10^–2^ Scm^–1^ prepared by the directional freezing method^4^. The upper and rear surfaces of the 3.45 mm thick CdTe wafer were formed by the cleavage planes. Two ohmic gold electrodes were applied by precipitating Au from a gold chloride solution to obtain a sandwich structure. Similar measurements were performed on a chlorine-doped n-type CdTe sample with a conductivity of 10 Scm^–1^. The 2 mm thick wafer was contacted with indium on both the top and bottom surfaces. For comparison, fluctuations were also measured on amorphous CdTe layers prepared by electrodeposition from a CdSO_4_ solution with a small amount of TeO_2_^5^. The structure of the layers was monitored with an X-ray diffractometer. The electrical conductivity of the 1–2 µm thick layers was 10^–4^ Scm^–1^.

The low-frequency current through the sample was measured with a Lock-In Stanford SR 810 narrowband amplifier. The current was calculated using a load resistor connected in series with the sample and the battery. The experiment was performed at room temperature on several samples of CdTe. The measured fluctuation current density* j*_*F*_ is shown in Fig. 2. Later, it will be shown that the peaks in the spectrum arise as a result of the interaction of the traps *t*_*i*_ with the valence band. Let us assume that a hole localized on a trap behaves like a particle in a potential well. The number of attempts to release it depends on the depth of the trap Δ*E*_*i*_ and frequency factor *ν*_*i*_ and appears in the signal of frequency *f*_*i*_ that we observe in the spectrum. The shallow traps are in thermal equilibrium with the band, when the number of excitations from the traps is equal to the number of captures on the traps^6^. Let’s say the mean lifetime of a hole at level *t*_*i*_ will be *τ*_*i.*_ The equality of release and capture of carriers for a given trap *t*_*i*_ is expressed by the relation.

1 $$\nu_{i} \exp ( - \Delta E_{i} /kT) \equiv f_{i} = \frac{1}{{\tau_{i} }},$$

The frequency factor *ν*_*i*_ = *N*_*V*_* uσ*_*i*_ is equal to the product of the effective density of states in the valence band (*N*_*V*_), the thermal velocity of holes (*u*), and, the effective cross-section for the capturing the hole (*σ*_*i*_), Δ*E*_*i*_ is the depth of the trap*, k* is the Boltzmann constant and *T* is the absolute temperature.

This equation also states, that the probability of a hole being excited from the trap into the band per second is equal to the probability of its retrapping per second. In the whole crystal, however, we assume a more general expression that applies to each *τ*_*i*_ and *f*_*i*_ from the interval of linear dependence of *j*_*F*_ on 1/*f*, namely

2 $$\tau = \frac{a}{f},$$

where *a* is a constant whose meaning is explained below. A similar expression was published by Hooge^7^ for noise power spectral density. In relation (5), will be shown that the mean value of the fluctuations is a linear function of 1/*f*. Processes of trapping of holes on the traps and their subsequent release lead to fluctuations of carriers concentration in the valence band, which we denote by Δ*p*(*f*)*.*

3 $$p(f) = p_{0} + \Delta p(f),$$

where *p*_*0*_ is the equilibrium concentration of holes unaffected by fluctuations, and *p* (*f*) is the total hole concentration.

As a result of fluctuations of the concentration, the current density *J* flowing through the crystalline CdTe sample is modulated by the current density *j*_*F*_ shown in Fig. 2.

 In the scheme in Fig. 2, the sample is supplied from a battery *B* with a direct current of density *J*. This current is modulated by an alternating current density *j*_*F*_ taken from the resistor *R*. Fluctuations taken from low noise resistor were amplified by a lock-in amplifier. Next, we derive the connection between the mean value of the fluctuation current and its frequency. The mean value of the fluctuation current density* j*_*F*_ is a function of the hole concentration Δ*p* (*f*):

4 $$\overline{{j_{F} (f)}} = e\overline{\Delta p(f)} \mu_{p} E = e\overline{{g\tau_{p} (f)}} \mu_{p} E,$$

where *e* is the elementary charge, *µ*_*p*_, and *τ*_*p*_ are the mobility and lifetime of the holes, respectively, *g* is the generation rate, and *E* is the electric field applied to the sample. A direct current *J* injects a charge into the sample of thickness *d* with a generation rate *g* = *J*/(*e* × *d*), as follows from solving the continuity equation while neglecting current losses. The product of the generation rate *g* and the carrier lifetime *τ* gives the carrier concentration Δ*p*. The voltage *V* across the sample creates an electric field *E* = *V*/*d*. After replacing *τ*_*p*_ according to expression (2), the generation rate *g* and the electric field *E* by appropriate relations given above, we get

5 $$\overline{{j_{F} (f)}} = \frac{JV}{{d^{2} }}\mu_{p} \frac{a}{f}.$$

**Figure 3:**
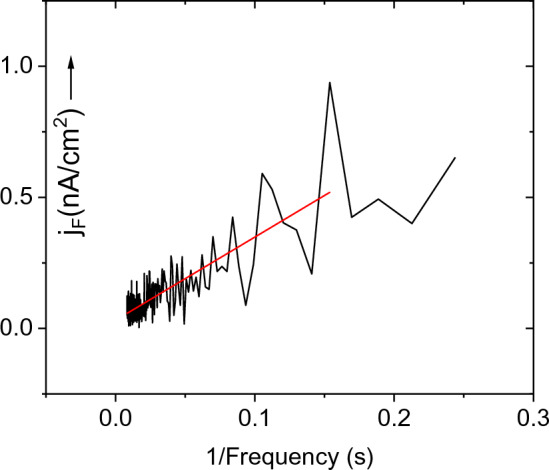
Dependence of the mean value of the fluctuation current density (straight line) on the inverse value of the frequency in the range of 4.0–112 Hz.

This means that with decreasing frequency, the mean value of the signal increases and is linear to 1/*f*, which is illustrated in the case of the semiconductor CdTe (See Fig. 3).

When traps from *N* unit cells in the crystal lattice interact with the band, the system behaves as a single cell, with each trap level having a concentration of *N*. As a result, the lifetime of current carriers relative to each trap is reduced by *N* times, and Eq. (1) is replaced by Eq. (2), where *a* = 1/*N*. The parameter *a* is a constant equal for all shallow levels in the investigated range.

**Figure 4:**
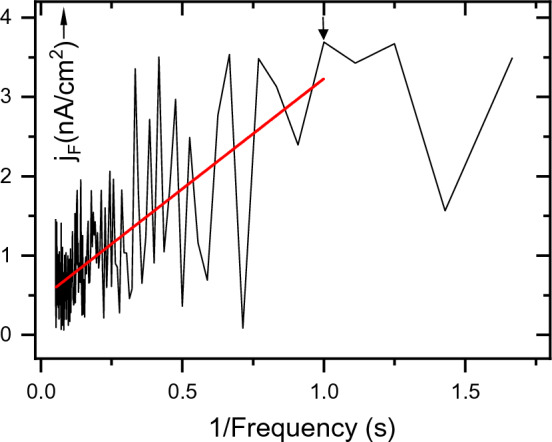
A measurement of the AC fluctuation current density in crystalline CdTe is shown ranging from 0.5 Hz to 19 Hz in steps of 0.1 Hz. The limiting value of the reciprocal of the frequency 1/*f*_*min*_, is indicated by an arrow in the spectrum and is equal to 1 Hz. The straight line shows the mean value of the fluctuation current density.

The balance between the shallow traps and the valence band leads to a linear dependence of the signal on the reciprocal of the frequency, unless it falls below *f*_*min*_ (See arrow in Fig. 4). The lowest frequency *f*_min_, which still lies on the 1/*f* line, determines the lifetime of the equilibrium carriers. For *f*_min_, the value of *τ* reaches a maximum equal to *τ*_max_, which actually is the stationary lifetime. Charges located in deeper potential wells are released after a longer time because the probability of their release is smaller. This situation, therefore, occurs at frequencies where the mean value of the signal deviates from the straight line.

**Figure 5:**
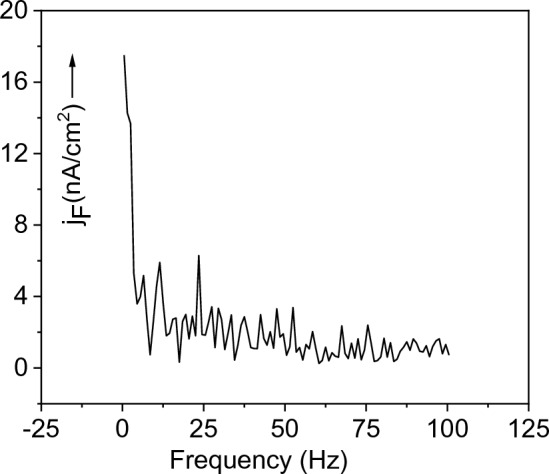
Fluctuation spectrum measured on amorphous CdTe. Compared to the spectrum in Fig. 2, it is not ordered and the individual peaks show larger amplitudes.

In Fig. 5 we present the fluctuation spectrum of amorphous CdTe measured on a layer deposited on a glass/ITO/CdS substrate for comparison with the spectrum of crystalline CdTe shown in Fig. 2. The absence of long-range periodicity and translation symmetry of the unit cells does not allow the creation of a hyperbolic course of the fluctuation amplitude nor a linear dependence of *j*_*F*_ on 1/*f.* Such fluctuations are not reproduced and cannot be evaluated.

## Results and discussion

The fluctuation of current density plotted against 1/*f* is shown in Fig. 3. From the slope of the fitted line, the product *µ*_*p*_*a* was determined using Eq. (5). We consider that the lowest frequency of the current, which still satisfies the linear dependence *j*_*F*_ (*f*), is decisive for the lifetime. In our case, the fitted line on the low-frequency side ends at 1 Hz (Fig. 4). For the value of *f*_*min*_ = 1 Hz, the product *µ*_*p*_*a/f*_*min*_ = *µ*_*p*_*τ*_*p*_ = (6.6 ± 0.3) × 10^–7^ cm^2^V^–1^ was obtained. 

We compare this result with the stationary values of mobility and lifetime. At the mobility *µ*_*p*_ = 86.5 cm^2^V^–1^ s^–1^^8^ we obtain the lifetime *τ*_p_ = 7.6 × 10^–9^ s. This value was compared with the lifetime calculated from the known diffusion length *L*_*p*_ of the minority carriers in n-type CdTe. Using *L*_*p*_ = 0.8 µm, published e.g. in Ref.^9^ and based on the relation *τ*_p_ = *L*_*p*_^2^/*D*_*p*_ where *D*_*p*_ = *µ*_*p*_* kT*/*e* is the diffusion coefficient for holes, we get the lifetime *τ*_p_ = 2.9 × 10^–9^ s which is in order of magnitude with the value calculated from the fluctuations.

The standard deviation of the extracted *µ*_*p*_*τ*_*p*_ value is 5%, which is mainly caused by the uncertainty in the slope of the 1/*f* graph.

Similarly, we obtained data for n-CdTe. The frequency spectrum was measured from 0.1 Hz to 20 Hz with a step of 0.1 Hz. The frequency *f*_*min*_ was 0.8 Hz. The product *µ*_*n*_*τ*_*n*_ was (7.5 ± 0.3) × 10^–8^ cm^2^V^–1^. At the mobility of 1176 cm^2^V^–1^ s^–1^^8^, the electron lifetime is 0.6 × 10^–10^ s. This electron lifetime was compared with the lifetime calculated from the diffusion length of electrons in p-type CdTe. We have determined its value of 0.6 µm by the evaluation of the surface photovoltage^10^. This method provides the diffusion length of the minority charge carriers-electrons. Using Einstein’s relation and the known mobility of electrons, the electrons` lifetime comes out 1.2 × 10^–10^ s in quite good agreement with the value obtained from low-frequency fluctuations.

## Conclusions

The charge carriers on the traps behave like particles in a potential well. For each shallow trap in equilibrium with the valence or conduction band, the equality between the rate of release and capture of charges applies, which can be expressed by the relation *τ* = 1/*f*. In the crystalline phase, there are unit cells that are characterized by translation symmetry and, consequently, have identical properties. When traps from N-unit cells in the crystal lattice interact with the band, the system behaves as a single cell, with each trap having a concentration of *N*. As a result, the lifetime of current carriers relative to each trap is reduced by *N* times, and Eq. (1) is replaced by Eq. (2), where *a* = 1/*N.* It leads to a decrease in the lifetime by a factor of *N*, so the resulting *τ* = 1/*Nf*, or *τ* = *a*/*f*, where *a* ≡ 1/*N*. Therefore, crystallinity leads not only to shortening the lifetime of the carriers but also to a reduction of the signal. The existence of a crystalline phase in a semiconductor manifests in a linear dependence of the mean value of the fluctuation current against 1/*f*, as shown in the case of crystalline CdTe. On the other hand, amorphous CdTe does not show linearity of the mean value of the signal against 1/*f.*

Analytical description (5) of the mean value of the fluctuations on 1/*f* enables the determination of the product *µa*. The lowest frequency *f*_min_, which still lies on the 1/*f* line, determines the lifetime of the equilibrium carriers. For *f*_min_, a maximum value of *τ*_max_ = *a*/*f*_min_ is obtained, which is the stationary lifetime. If *f* < *f*_min_, the fluctuations are related to traps that are not in equilibrium with any band. Our model was applied to the p-CdTe crystalline semiconductor and the mobility-lifetime product *µ*_*p*_*τ*_*p*_ = (6.6 ± 0.3) × 10^–7^ cm^2^ V^–1^ was obtained. For similarly studied n-type CdTe *µ*_*n*_*τ*_*n*_ = (7.5 ± 0.3) × 10^–8^ cm^2^V^–1^was found.

Given that the results were obtained based on the validity of relation (1), which expresses the stationarity of the interaction process of shallow traps with the valence band, the evaluated parameter *µτ* can be considered a stationary quantity. In contrast, the authors^11–14^, who deal with the transport of the injected charge, achieve higher *µτ* values. This is mainly due to the effect of the traps, which are not shallow, and which in these non-stationary conditions slowly release charge after injection, thereby increasing *τ* and, consequently, the product *µτ*. In addition, charge transport is most affected by the slowest relaxations from the deepest traps. Similar measurements on similar CdTe samples have been performed in Ref.^11^, where the *µτ* product of the order of 10^–3^ cm^2^V^–1^ is in agreement with typical values measured by the non-stationary methods.

For the function of radiation detectors, the product *µτ* obtained on the basis of non-stationary transport measurements is perhaps more suitable, since the influence of traps cannot be excluded during detection. The disadvantage is that it is necessary to take into account the actual profile of the electric field in the sample. Neglecting the sample`s polarization can lead to underestimation of *µτ*. From this point of view, the results of our stationary measurement have a certain advantage; they correspond to an ideal material—without traps.

The model described here elucidates the processes that lead to current fluctuations in semiconductors with a 1/*f* spectrum and at the same time allows their use to determine the product *µτ*. This product is an important parameter that determines the performance of electronic and photonic devices.

## Data Availability

The data that support the findings of this study are available from the corresponding author upon reasonable request.
